# Pulmonary Coinfection of *Pneumocystis jirovecii* and *Aspergillus* Species

**DOI:** 10.1093/ofid/ofaf018

**Published:** 2025-01-13

**Authors:** Stefan Hatzl, Christina Geiger, Lisa Kriegl, Andreas Reinisch, Albert Wölfler, Georg Apfaltrer, Markus Keldorfer, Siegfried Rödl, Martin Hoenigl, Philipp Eller, Robert Krause

**Affiliations:** Department of Internal Medicine, Intensive Care Unit, Medical University of Graz, Graz, Austria; BioTechMed-Graz, Graz, Austria; Division of Infectious Diseases, Department of Internal Medicine, Medical University of Graz, Graz, Austria; BioTechMed-Graz, Graz, Austria; Division of Infectious Diseases, Department of Internal Medicine, Medical University of Graz, Graz, Austria; Division of Hematology, Department of Internal Medicine, Medical University of Graz, Graz, Austria; Department of Blood Group Serology and Transfusion Medicine, Medical University of Graz, Graz, Austria; Division of Hematology, Department of Internal Medicine, Medical University of Graz, Graz, Austria; Division of Pediatric Radiology, Department of Radiology, Medical University of Graz, Graz, Austria; Department of Pediatrics and Adolescent Medicine, Pediatric Intensive Care Unit, Medical University of Graz, Graz, Austria; Department of Pediatrics and Adolescent Medicine, Pediatric Intensive Care Unit, Medical University of Graz, Graz, Austria; BioTechMed-Graz, Graz, Austria; Division of Infectious Diseases, Department of Internal Medicine, Medical University of Graz, Graz, Austria; Department of Internal Medicine, Intensive Care Unit, Medical University of Graz, Graz, Austria; BioTechMed-Graz, Graz, Austria; Division of Infectious Diseases, Department of Internal Medicine, Medical University of Graz, Graz, Austria

**Keywords:** aspergillosis, immunocompromised patients, invasive pulmonary aspergillosis, PCP-IPA coinfection, *Pneumocystis jirovecii*

## Abstract

In this multicenter study of 387 patients who were immunocompromised, 4.5% with invasive pulmonary aspergillosis also had *Pneumocystis jirovecii* pneumonia. Predictors of coinfection included elevated β-D-glucan and prolonged corticosteroid use. Coinfection correlated with reduced 30-day survival (22% vs 57%), suggesting that early identification and prophylaxis may improve outcomes.

## Graphical Abstract

**Graphical abstract:**
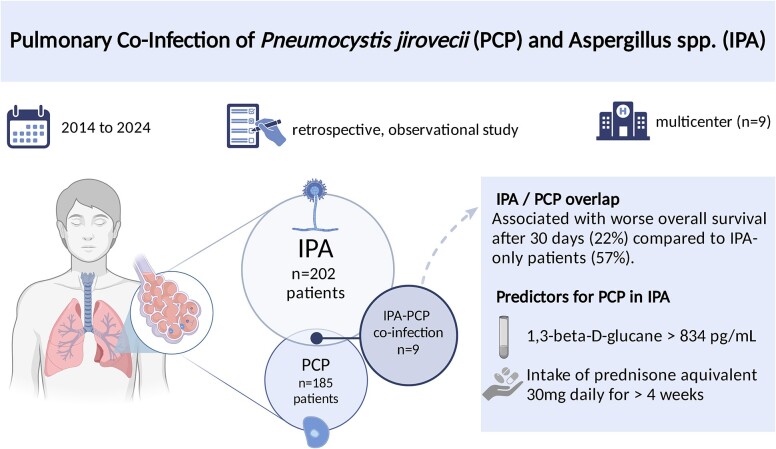

*Pneumocystis jirovecii* causes *Pneumocystis* pneumonia (PCP), a serious opportunistic infection affecting individuals who are HIV infected and others who are immunocompromised. While PCP was once a common AIDS-defining illness, its incidence has dropped in HIV-positive populations due to antiretroviral therapy and prophylaxis. However, PCP cases are rising among patients who are not HIV immunocompromised, who face higher mortality rates (24%–67%) as compared with patients with HIV (7%–17%), often due to delayed treatment and complications such as hypoxemia and coinfections [1].

Invasive pulmonary aspergillosis (IPA), another fungal infection affecting a similar immunocompromised population, rarely co-occurs with PCP, though a few case reports link this coinfection to prolonged high-dose glucocorticoid use without adequate prophylaxis. This systematic multicenter study examines the frequency, clinical outcomes, and treatment of PCP coinfection in patients with IPA [2–4].

## METHODS

### Patient Cohort

We conducted a retrospective multicenter cohort study at 9 treatment centers, including all adult patients (age ≥18 years) diagnosed with IPA and PCP from 1 January 2014 to 1 June 2024. Patient data were collected uniformly as previously described [5]. The study received ethics approval (EK:32-302ex19/20) and adhered to the Declaration of Helsinki principles.

### Definition of IPA and PCP

IPA was defined per the updated EORTC-MSG criteria (European Organisation for Research and Treatment of Cancer/Mycoses Study Group) or, for patients in the intensive care unit (ICU) lacking EORTC host factors, the FUNDICU criteria (Invasive Fungal Diseases in Adult Patients in Intensive Care Unit). For cases missing both sets of criteria, modified ASP-ICU criteria (aspergillosis in intensive care units) were applied, bypassing the dependency on specific host factors. PCP was defined according to the EORTC-MSG criteria [2, 4, 6].

### Statistical Analysis

Statistical analyses were conducted with Stata version 16 (StataCorp LLC). Descriptive statistics summarized baseline characteristics, applying χ^2^ or Fisher exact test for categorical variables and Wilcoxon rank sum tests for continuous variables. Univariate logistic regression and adaptive logistic least absolute shrinkage and selection operator (LASSO) regression identified predictors of baseline characteristics and factors linked to IPA-PCP coinfection [7]. The Youden index determined optimal cutoff points for continuous predictors.

Thirty-day overall survival (OS) post–IPA diagnosis was estimated by Kaplan-Meier curves, with group comparisons via the log-rank test. Univariable and multivariable Cox proportional hazards models identified independent predictors of 30-day OS, adjusting survival time by 1 day for patients who died on the diagnosis day (n = 4). Statistical significance was set at *P* < .05.

## RESULTS

### *P jirovecii* Pneumonia in IPA

Since it was unclear whether PCP or IPA was the initial infection, we analyzed an IPA cohort (n = 202) and a PCP cohort (n = 185) from 2 perspectives, examining the overlap and clinical characteristics to clarify the sequence of detecting the infection. Nine patients met diagnostic criteria for both infections, designated as having IPA-PCP coinfection (Supplementary Figure 1). In these cases, further bronchoalveolar lavage (BAL) diagnostics were prompted by either a positive *Aspergillus* sputum culture result (n = 6) or a serum galactomannan (GM) with an optical density index ≥0.5 (n = 3). Serum GM was positive at a median 2 days (IQR, 1–3) and sputum culture at a median 3 days (1–4) before bronchoscopy, leading to PCP diagnosis.

Focusing on the IPA cohort, we examined the relationship between early signs of invasive mold infection and subsequent PCP. *P jirovecii* polymerase chain reaction in BAL fluid was performed in 167 of 202 (82%) patients with IPA, while all patients in the PCP cohort had at least 1 *Aspergillus* biomarker (BAL-GM, serum GM, or culture from BAL fluid).

### Cohort Description

Of the 202 patients diagnosed with IPA, 9 (4.5%) met the criteria for IPA-PCP coinfection. The median (IQR) age of the IPA cohort was 63 years (54–71), and patients with IPA-PCP were significantly older with a median age of 67 years (65–76). A total of 63 patients (31%) were female, with similar gender distribution between IPA and IPA-PCP groups. Baseline laboratory characteristics, including blood counts and renal and liver function, showed no significant differences.

All patients with IPA-PCP had an EORTC-MSG risk factor: 8 were undergoing long-term high-dose glucocorticoids while 1, an allogeneic stem cell transplant recipient for relapsing acute myeloid leukemia, did not receive glucocorticoids at diagnosis. None of the patients with IPA-PCP had received antimold or anti-*Pneumocystis* prophylaxis. Overall, 155 patients (77%) required ICU admission due to critical illness, with no significant ICU characteristic differences between the IPA and IPA-PCP groups (Supplementary Table 1). Of the 9 patients with IPA-PCP coinfection, 5 underwent autopsy, with IPA confirmed histopathologically in all cases. Additional mycologic criteria are summarized in Supplementary Table 2.

### Predictors of IPA-PCP Coinfection

Univariable logistic regression identified age as a significant predictor for IPA-PCP coinfection, with a 2.3-fold increased risk per 10-year increment (odds ratio [OR], 2.3; 95% CI, 1.1–5.0; *P* = .01). Body mass index showed a borderline association (OR, 1.6; 95% CI, 1.0–2.5; *P* = .07), while leukocyte count was also significantly associated with coinfection (OR, 1.3; 95% CI, 1.0–1.7; *P* = .03). Among mycologic markers, β-D-glucan was strongly associated with an increased risk (OR, 1.3; *P* < .001), whereas variables such as GM and BAL culture were not significant. Corticosteroid use emerged as a particularly strong predictor of IPA-PCP coinfection (OR, 27.0; *P* < .001; Supplementary Table 3).

In a multivariable context-adaptive LASSO regression, analysis identified β-D-glucan (OR, 1.3; *P* < .001) as the sole predictor of IPA-PCP coinfection. The optimal discriminatory cutoff for β-D-glucan, based on the Youden index, was 834 pg/mL (Supplementary Figure 2).

### IPA-PCP Coinfection Affects 30-Day OS

**Figure 1.**
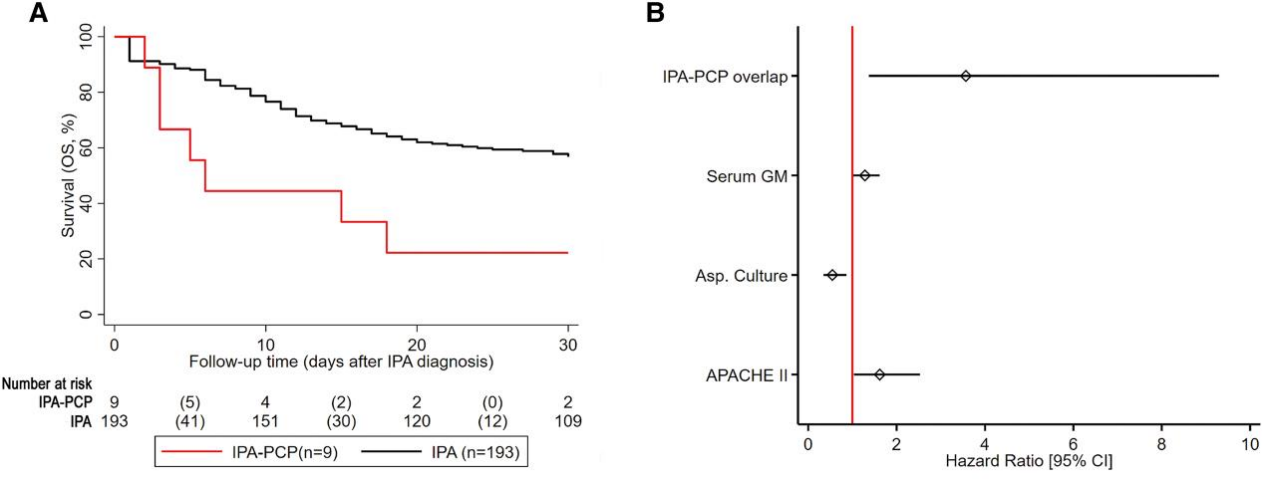
Survival analysis of patients with IPA-PCP coinfection. *A*, Kaplan-Meier plot comparing survival rates of patients with IPA-PCP coinfection with those with IPA alone. *B*, Results from the multivariable Cox regression model in a forest plot for enhanced readability. Abbreviations: APACHE II, Acute Physiology and Chronic Health Evaluation II; Asp, aspergillus; GM, galactomannan; IPA, invasive pulmonary aspergillosis; PCP, *Pneumocystis* pneumonia.

In the crude analysis, IPA-PCP coinfection was linked to significantly worse 30-day OS as compared with IPA alone (log-rank *P* = .01; Figure 1*A*). The 30-day OS rates at days 5, 15, and 30 were 88% (95% CI, 83%–92%), 68% (61%–74%), and 57% (49%–63%) for the IPA group vs 56% (20%–80%), 33% (8%–62%), and 22% (4%–52%) for the IPA-PCP group.

To determine whether *P jirovecii* coinfection independently predicted 30-day OS, we conducted univariable Cox regression to identify survival-related factors (Supplementary Table 4). After excluding nested variables (leukocyte count, platelet decrease, bilirubin, and creatinine, which contribute to the APACHE II score [Acute Physiology and Chronic Health Evaluation II]), we incorporated all variables associated with impaired survival into a multivariable Cox regression model. This analysis confirmed IPA-PCP coinfection as a strong independent predictor of reduced 30-day OS, with a multivariable hazard ratio of 3.6 (95% CI, 1.4–9.3; Figure 1*B*).

### Clinical Findings and Management of IPA-PCP Coinfection

All 9 patients with IPA-PCP coinfection were diagnosed with probable IPA antemortem according to the EORTC-MSG criteria. Of these, 7 patients died and 6 underwent necropsy, with histopathologic confirmation of invasive aspergillosis in 5 cases. *P jirovecii* was detected in BAL fluid by polymerase chain reaction in all 9 patients, supporting a probable PCP diagnosis (postmortem *Pneumocystis* staining was unavailable).

Radiologically, all patients with IPA-PCP coinfection presented with extensive bilateral peripheral ground-glass opacities and the tree-in-bud sign (Supplementary Figure 3). Treatment included voriconazole for 7 patients, with 2 receiving either anidulafungin or caspofungin for IPA at the discretion of the treating physician. All 9 patients received trimethoprim-sulfamethoxazole for PCP.

## DISCUSSION

In our study, IPA-PCP coinfection was rare, occurring in 4.5% of patients with IPA. The sequence of infection remains unclear, complicating the identification of appropriate investigative directions for either the IPA or PCP cohort. We chose to explore coinfection through the IPA cohort, finding that the incidence was consistent, with 9 of 202 IPA cases (4.5%) and 9 of 185 PCP cases (4.9%).

Previous reports of IPA-PCP coinfection have been anecdotal, consistently noting long-term high-dose glucocorticoid use as a risk factor [3]. Our findings align with this, as all but 1 of the IPA-PCP coinfection cases had received glucocorticoids, with most patients (89%) receiving doses exceeding 30 mg of prednisone for >4 weeks, which is a known risk factor for both infections. Among the most immunocompromised cases in the EORTC-MSG category, the incidence of coinfection rose to approximately 12% (9/78). This emphasizes the need for heightened awareness of IPA-PCP coinfection in this vulnerable population. Notably, none of the patients received prophylaxis for either IPA or PCP, possibly due to a lack of awareness, toxicity concerns, or patient compliance issues [2, 8, 9].

Radiologically, all IPA-PCP coinfection cases presented with extensive bilateral ground-glass opacities and a tree-in-bud pattern on high-resolution chest computed tomography scans. While these findings are common to both infections, their sensitivity and specificity for distinguishing between them are limited. Nevertheless, this radiologic pattern may suggest a higher suspicion for coinfection [10, 11].

LASSO regression analysis identified elevated β-D-glucan levels as an independent predictor of IPA-PCP coinfection. Specifically, β-D-glucan has been validated as a useful screening tool for PCP. A β-D-glucan threshold of 834 pg/mL may warrant further PCP examination in patients with IPA, as our data showed significantly higher levels in those with coinfection. However, this threshold should be interpreted with caution due to potential assay variability, and clinicians should not dismiss *P jirovecii* infection at lower levels, especially in patients with IPA experiencing acute respiratory failure under strong immunosuppression [12, 13].

Recent epidemiologic trends indicate a rise in PCP incidence and related mortality, with a shift in underlying conditions from HIV and hematologic malignancies to glucocorticoid-induced immunosuppression, a trend reflected in our IPA-PCP coinfection cases [14].

In conclusion, we identified IPA-PCP coinfection in about 5% of all patients with IPA and 12% among those with severe immunosuppression, primarily due to long-term glucocorticoid therapy. Our findings suggest that high β-D-glucan levels are strong predictors of PCP in patients with IPA, with coinfection associated with poor survival outcomes, necessitating further research into prevention and early treatment strategies.

## Supplementary Material

ofaf018_Supplementary_Data

## Supplementary Data

Supplementary materials are available at *Open Forum Infectious Diseases* online. Consisting of data provided by the authors to benefit the reader, the posted materials are not copyedited and are the sole responsibility of the authors, so questions or comments should be addressed to the corresponding author.

## Notes

***Author contributions*.** S. H. and R. K. designed the study. S. H. wrote the first draft. S. H. performed the biometry. S. H. and R. K. interpreted the results. S. H. and R. K. collected the data. All authors read and revised the manuscript.

***Data availability*.** The data set used and analyzed during the current study is available from the first author on reasonable request.

***Financial support*.** This research did not receive any funding.
