# Correction to: Pulmonary Coinfection of *Pneumocystis jirovecii* and *Aspergillus* Species

**DOI:** 10.1093/ofid/ofaf083

**Published:** 2025-02-17

This is a correction to: Stefan Hatzl, Christina Geiger, Lisa Kriegl, Andreas Reinisch, Albert Wölfler, Georg Apfaltrer, Markus Keldorfer, Siegfried Rödl, Martin Hoenigl, Philipp Eller, Robert Krause, Pulmonary Coinfection of *Pneumocystis jirovecii* and *Aspergillus* Species, *Open Forum Infectious Diseases*, Volume 12, Issue 2, February 2025, ofaf018, https://doi.org/10.1093/ofid/ofaf018

The graphical abstract originally included as part of this article was unrelated and has been replaced online.

