# Supplementary material for: Pulmonary Coinfection of Pneumocystis jirovecii and Aspergillus Species
Source: Open Forum Infect Dis. 2025 Jan 13;12(2):ofaf018. doi: 10.1093/ofid/ofaf018 (PMC11786051; doi:10.1093/ofid/ofaf018)
Supplement: ofaf018_Supplementary_Data [file ofaf018_supplementary_data.docx]

**Supplementary**

**Pulmonary Co-Infection of Pneumocystis jirovecii and Aspergillus species**

***Hatzl S et al.***


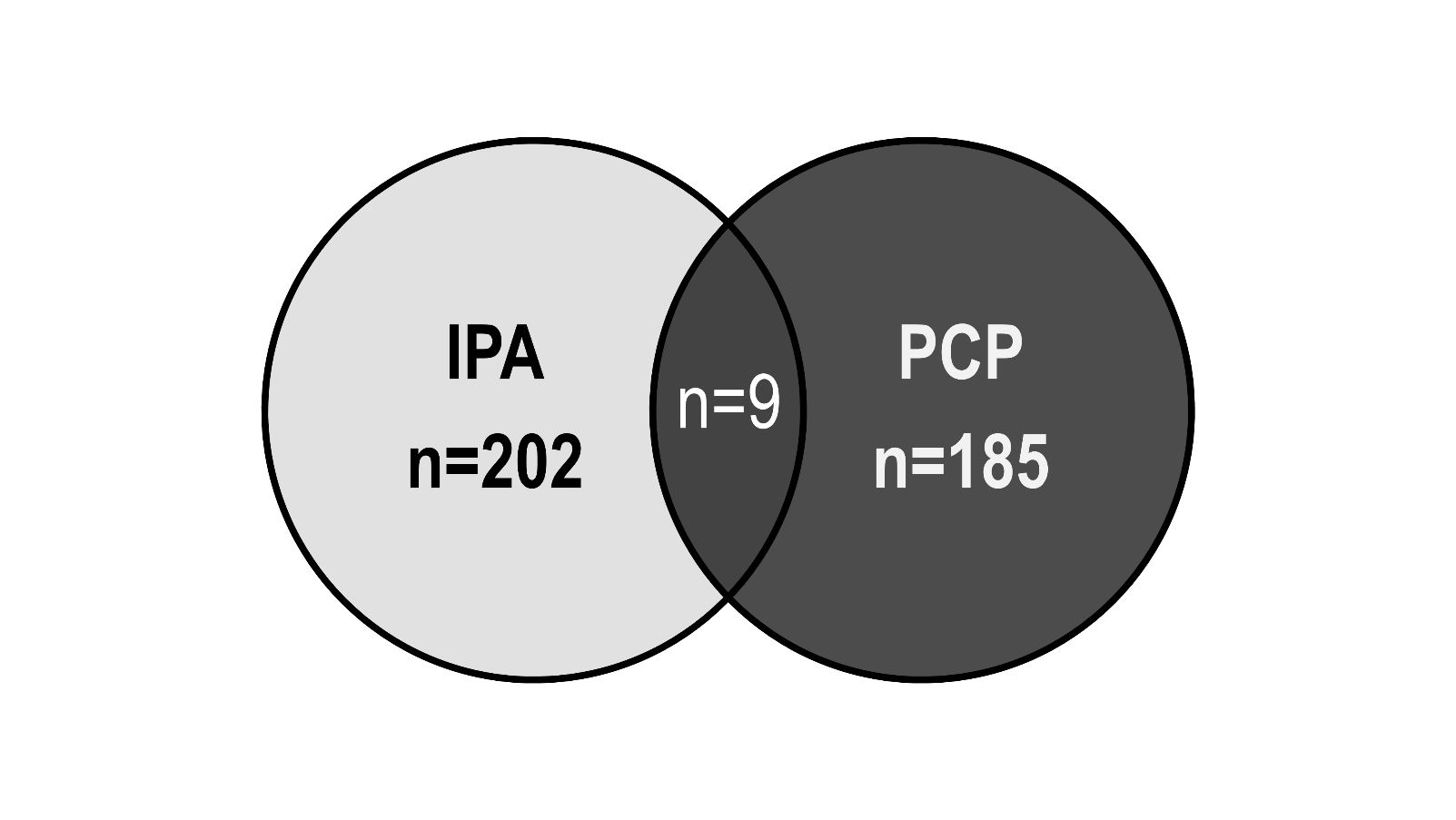
**Supplementary Figure 1:**

**Supplementary Figure 1:** This Venn diagram shows two patient cohorts: those with invasive pulmonary aspergillosis (IPA) and those with *Pneumocystis jirovecii* pneumonia (PCP). Nine patients had co-infection.

**Supplementary Figure 2:**


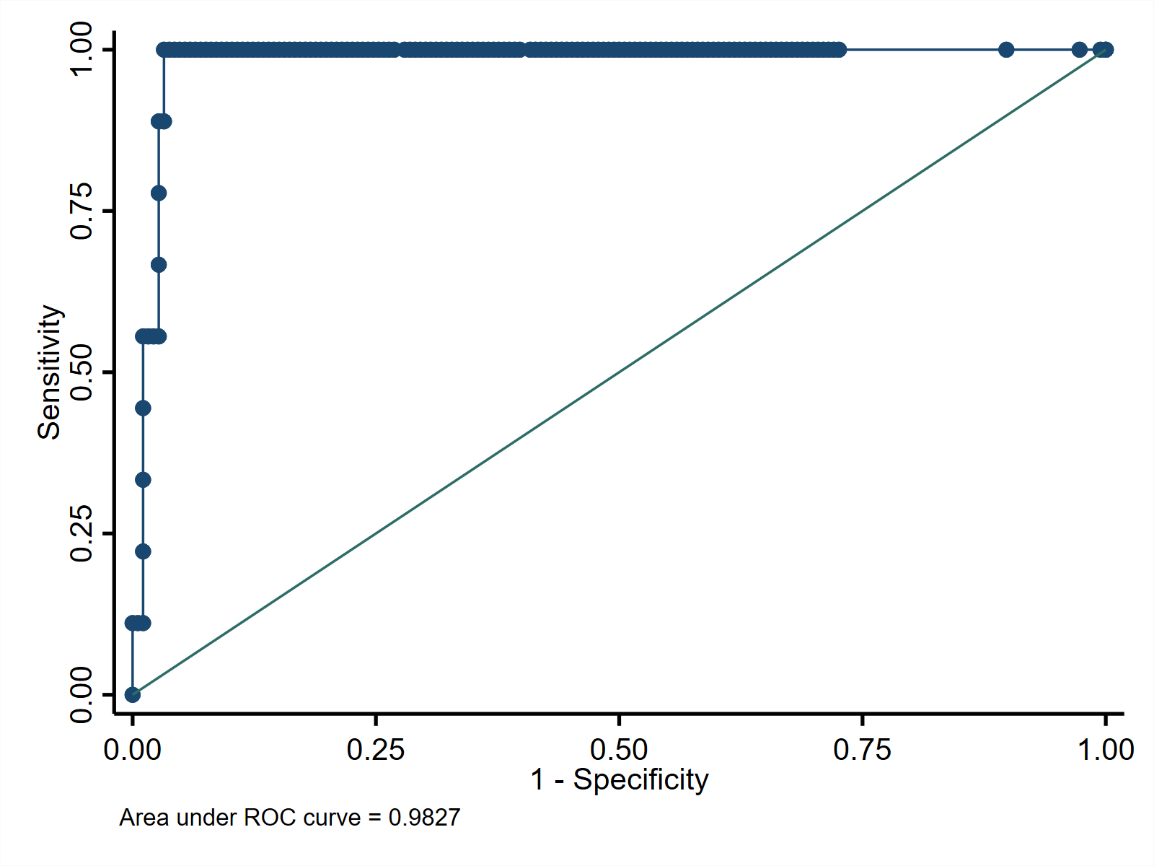


**Supplementary Figure 2:** Receiver Operating Characteristic (ROC) curve illustrating the diagnostic performance of *β*-D-glucan levels in predicting co-infection. The optimal discriminatory cut-off, determined by Youden's index, is indicated at 834 pg/mL. The area under the curve (AUC) reflects the overall accuracy of *β*-D-glucan as a predictive biomarker for co-infection.


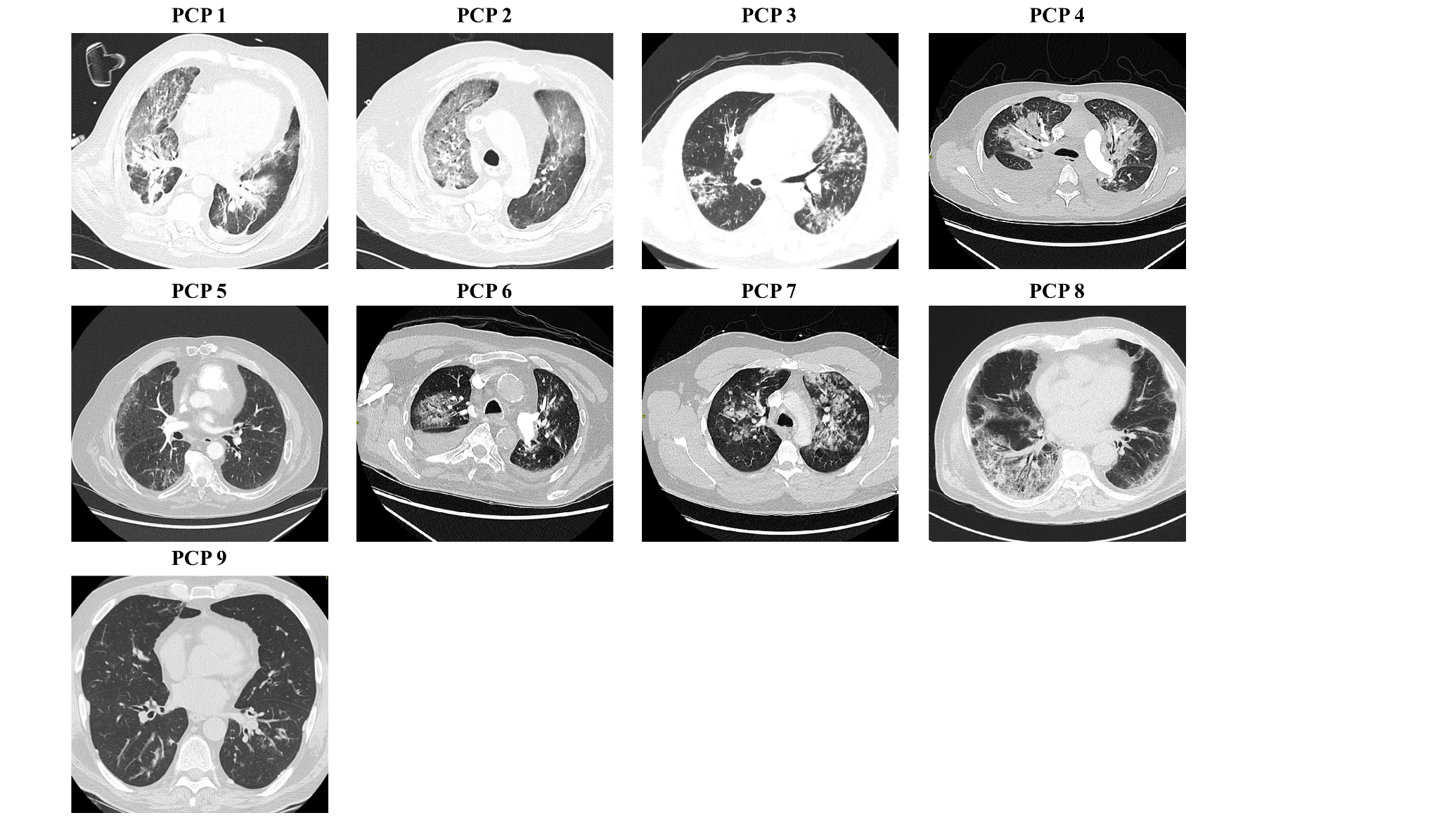
**Supplementary Figure 3:**

**Supplementary Figure 3:** Representative high-resolution computed tomography scans from all patients with IPA-PCP co-infection.

**Supplementary Table 1:**

| **Variable** | **Missing n (%)** | **Overall (n=202)** | **IPA (n=193)** | **IPA-PCP (n=9)** | **p-value** |
| --- | --- | --- | --- | --- | --- |
| Age (years) | 0 (0%) | 63 [54-71] | 61 [53-70] | 67 [65-76] | **0.02** |
| Females | 0 (0%) | 63 (31%) | 60 (31%) | 3 (33%) | 0.88 |
| BMI (kg/m²) | 0 (0%) | 25.4 [22.7-28.4] | 25.3 [22.6-28.4] | 25.8 [25.4-27.7] | 0.17 |
|  |  |  |  |  |  |
| **Laboratory findings** |  |  |  |  |  |
| Leukocytes [G/L] | 0 (0%) | 9.4 [4.6-14.6] | 9.4 [4.6-13.8] | 9.6 [7.1-15.1] | 0.50 |
| Neutrophils [G/L] | 0 (0%) | 8.0 [3.2-12.0] | 7.9 [3.2- 12.0] | 9.1 [5.8-14.0] | 0.60 |
| Lymphocytes [G/L] | 0 (0%) | 0.6 [0.3-1.0] | 0.6 [0.3-1.0] | 0.6 [0.3-0.9] | 0.88 |
| Haemoglobin [g/dL] | 0 (0%) | 9.8 [8.9-11.8] | 9.8 [8.9-12.0] | 9.9 [9.3-10.3] | 0.66 |
| Platelets [G/L] | 0 (0%) | 149 [67-235] | 151 [65-231] | 128 [101-245] | 0.90 |
| CRP [mg/L] | 0 (0%) | 116 [53-201] | 114 [53-200] | 159 [39-232] | 0.80 |
| PCT [ng/ml] | 21 (10%) | 0.9 [0.3-4.0] | 0.8 [0.3-4.1] | 1.09 [0.6-2.1] | 0.67 |
| Bilirubin [mg/dL] | 0 (0%) | 0.7 [0.4-1.8] | 0.7 [0.4-1.8] | 0.8 [0.5-1.7] | 0.86 |
| Creatinine [mg/dL] | 0 (0%) | 1.3 [0.8-7.0] | 1.3 [0.8-7.0] | 2.1 [0.9-7.0] | 0.35 |
|  |  |  |  |  |  |
| **Mycological findings** |  |  |  |  |  |
| Serum GM (ODI) | 10 (5%) | 0.51 [0.15-0.95] | 0.53 [0.16-0.96] | 0.15 [0.12-0.65] | 0.30 |
| BAL GM (ODI) | 43 (22 %) | 4.29 [1.75-7.38] | 4.48 [1.75-7.45] | 2.91 [1.89-6.82] | 0.49 |
| *β*-D-Glucan pg/mL | 11 (5%) | 92 [15-263] | 85 [15-335] | 1703 [1061-2262] | **<0.001** |
| Asp-PCR | 134 (66%) | 47 (23%) | 44 (23%) | 3 (33%) | 0.75 |
| Asp-LFD | 139 (70%) | 40 (20%) | 38 (20%) | 2 (22%) | 0.47 |
| BAL-Culture | 35 (17%) | 118 (58%) | 113 (58) | 5 (55%) | 0.21 |
| *Aspergillus fumigatus* | / | 110 (54%) | 106 (54%) | 4 (44%) | / |
| *Aspergillus flavus* | / | 1 (1%) | 1 (1%) | 0 (0%) | / |
| *Aspergillus calidostus* | / | 1 (1%) | 1 (1%) | 0 (0%) | / |
| *Aspergillus niger* | / | 4 (4%) | 4 (3%) | 0 (0%) | / |
| *Aspergillus terreus* | / | 2 (1%) | 1 (1%) | 1 (11%) | / |
|  |  |  |  |  |  |
| **Immunosuppression** |  |  |  |  |  |
| EORTC – risk factor | 0 (0%) | 78 (39%) | 69 (36%) | 9 (100%) | **<0.001** |
| Neutropenia | 0 (0%) | 30 (15%) | 28 (15%) | 2 (22%) | 0.46 |
| Haematological Malignancy | 0 (0%) | 41 (21%) | 39 (20%) | 2 (22%) | 0.89 |
| Corticoids | 0 (0%) | 51 (25%) | 43 (22%) | 8 (89%) | **<0.001** |
|  |  |  |  |  |  |
| **Anti-Fungal prophylaxis** | 100 (0%) | / | / | / | **/** |
| Anti-Mold | / | 24 (12%) | 24 (13%) | 0 (0%) | 0.25 |
| Anti-PCP | / | 26 (13%) | 26 (14%) | 0 (0%) | 0.21 |
|  |  |  |  |  |  |
| **ICU-Characteristics** |  |  |  |  |  |
| ICU admission | 0 (0%) | 155 (77%) | 148 (77%) | 7 (78%) | 0.94 |
| APACHE II score | 0 (0%) | 26 [19-33] | 26 [19-33] | 31 [20-35] | 0,45 |
| SOFA | 0 (0%) | 7 [5-10] | 7 [4-10] | 7 [5-9] | 0.87 |
| paO_2_/FiO_2_ | 0 (0%) | 103 [71-148] | 103 [74-146] | 62 [52-167] | 0.38 |
| Ventilatory support | 0 (0%) | 155 (100%) | 148 (100%) | 7 (100%) | 0.65 |
| *HNFC* | 0 (0%) | 3 (2%) | 3 (2%) | 0 (0%) | / |
| *NIV* | 0 (0%) | 14 (9%) | 14 (10%) | 0 (0%) | / |
| *IV* | 0 (0%) | 114 (74%) | 109 (73%) | 5 (71%) | / |
| *vv-ECMO* | 0 (0%) | 24 (15%) | 22 (15%) | 2 (29%) | / |

**Supplementary Table 1**: Baseline characteristics of patients with IPA or IPA-PCP co-infection.

BMI – body mass index; CRP – C-reactive protein; PCT – procalcitonin; GM – galactomannan; Asp – *Aspergillus* spp.; LFD – lateral flow device; BAL – bronchoalveolar lavage; EORTC – European Organisation for Research and Treatment of Cancer/Mycoses Study Group; PCP – *Pneumocystis jirovecii* pneumonia; ICU – intensive care unit; APACHE – Acute Physiology and Chronic Health Evaluation II; SOFA – Sequential Organ Failure Assessment; HFNC – high-flow nasal cannula; NIV – non-invasive ventilation; IV – invasive ventilation; vv-ECMO – veno-venous extracorporeal membrane oxygenation.

| **ID** | **Chronic condition** | **Sputum Culture** | **BAL culture** | **SER-GM** | **BAL-GM** | **Asp-PCR** | **Asp-LFD** | **Autopsy** | **Proven** |
| --- | --- | --- | --- | --- | --- | --- | --- | --- | --- |
| PCP 1 | Meningioma |  | *Asp. terreus* |  | 1.07 | + | - | yes | yes |
| PCP 2 | Cryptogenic organizing pneumonia | *Asp. fumigatus* |  | 0.39 | 2.41 |  |  | yes | yes |
| PCP 3 | Systemic lupus erythematosus | *Asp. fumigatus* | *Asp. fumigatus* | 0.12 | 10.63 | + | + | no |  |
| PCP 4 | PR3-ANCA-associated vasculitis |  | *Asp. fumigatus* | 7.76 | 3.76 | + | + | no |  |
| PCP 5 | Psoriatic arthritis | *Asp. fumigatus* |  | 0.19 | 6.82 |  |  | yes | yes |
| PCP 6 | sAML |  |  | 0.9 | 0.35 | - | - | yes | yes |
| PCP 7 | Inflammatory cardiomyopathy | *Asp. fumigatus* | *Asp. fumigatus* | 0.12 | 2.00 |  |  | yes | yes |
| PCP 8 | Splenic marginal zone lymphoma | *Asp. fumigatus* | *Asp. fumigatus* | 0.10 | 7.12 |  |  | no |  |
| PCP 9 | CMML | *Asp. fumigatus* |  | 0.11 | 1.89 |  |  | no |  |

**Supplementary Table 2**: Mycological characteristics of the IPA-PCP co-infection patients

ID – unique patient identifier; Asp – Aspergillus, BAL – broncho alveolar lavage; SER – serum, PCR -polymerase chain reaction; LFD – lateral flow device; sAML – secondary acute myeloid leukemia, CMML – chronic myelomonocytic leukemia

| **Variable** | **n** | **OR [95%CI]** | **p-value** |
| --- | --- | --- | --- |
| Age per 10 years | 202 | 2.3 [1.1-5.0] | **0.01** |
| Female | 202 | 0.9 [0.2-3.7] | 0.89 |
| BMI per 5 kg/m² | 202 | 1.6 [1.0-2.5] | 0.07 |
|  |  |  |  |
| **Laboratory findings** |  |  |  |
| Leukocytes per 5 G/L | 202 | 1.3 [1.0-1.7] | **0.03** |
| Neutrophils per 5 G/L | 202 | 1.2 [1.0-1.4] | 0.06 |
| Lymphocytes G/L | 202 | 1.0 [0.8-1.3] | 0.89 |
| Haemoglobin per 5g/dL | 202 | 0.5 [0.1-2.6] | 0.39 |
| Platelets per 50 G/L | 202 | 0.9 [0.7-1.3] | 0.81 |
| CRP per 50 mg/L | 202 | 1.1 [0.8-1.4] | 0.70 |
| PCT per 10 ng/ml | 181 | 1.2 [1.0-1.4] | 0.09 |
| Bilirubin [mg/dL] | 202 | 0.9 [0.7-1.2] | 0.47 |
| Creatinine [mg/dL] | 202 | 1.1 [0.9-1.4] | 0.37 |
|  |  |  |  |
| **Mycological findings** |  |  |  |
| Serum GM (ODI) | 192 | 1.1 [0.7-1.6] | 0.67 |
| BAL GM (ODI) | 152 | 0.9 [0.8-1.2] | 0.53 |
| *β*-D-Glucan per 100 pg/mL | 195 | 1.3 [1.2-1.4] | **<0.001** |
| Asp-PCR | 68 | 1.4 [0.1-14.0] | 0.80 |
| Asp-LFD | 61 | 0.5 [0.1-3.8] | 0.51 |
| BAL-Culture | 199 | 0.9 [0.2-3.3] | 0.82 |
|  |  |  |  |
| **Immunosuppression** |  |  |  |
| Neutropenia | 202 | 1.7 [0.3-8.5] | 0.53 |
| Haematological Malignancy | 202 | 1.1 [0.2-5.6] | 0.83 |
| Corticoids | 202 | 27.0 [3.3-222] | **<0.001** |
|  |  |  |  |
| **ICU-characteristics** |  |  |  |
| ICU admission | 202 | 1.1 [0.2-5.3] | 0.93 |
| APACHEII | 202 | 1.0 [0.9-1.1] | 0.64 |

**Supplementary Table 3:**

**Supplementary Table 3:** Univariable predictors of IPA-PCP co-infection.

Variables include demographic, laboratory, mycological, immunosuppression, and ICU-related characteristics assessed for their association with IPA-PCP co-infection. Odds ratios (OR) with 95% confidence intervals (CI) and p-values are provided. **Significant predictors** include age, leukocyte count, β-D-glucan levels, and corticosteroid use.

BMI – body mass index; CRP – C-reactive protein; PCT – procalcitonin; GM – galactomannan; ODI – optical density index; BAL – bronchoalveolar lavage; Asp-PCR – *Aspergillus* polymerase chain reaction; Asp-LFD – *Aspergillus* lateral flow device; APACHE II – Acute Physiology and Chronic Health Evaluation II score; ICU – intensive care unit.

**Supplementary Table 4:**

| **Variable** | **n** | **HR [95%CI]** | **p-value** |
| --- | --- | --- | --- |
| IPA-PCP co-infection | 202 | 2.8 [1.3-6.0] | **0.01** |
|  |  |  |  |
| Age per 10 years | 202 | 1.0 [0.9-1.2] | 0.90 |
| Female | 202 | 0.9 [0.6-1.4] | 0.74 |
| BMI per 5 kg/m² | 202 | 1.0 [0.8-1.3] | 0.75 |
|  |  |  |  |
| **Laboratory findings** |  |  |  |
| Leukocytes per 5 G/L increase | 202 | 1.1 [1.0-1.3] | **0.05** |
| Neutrophils per 5 G/L increase | 202 | 1.1 [0.9-1.1] | 0.28 |
| Lymphocytes per 5 G/L increase | 202 | 1.0 [0.7-1.6] | 0.91 |
| Haemoglobin per 5g/dL increase | 202 | 0.9 [0.6-1.5] | 0.72 |
| Platelets per 50 G/L decrease | 202 | 1.2 [1.1-1.3] | **<0.001** |
| CRP per 100 mg/L increase | 202 | 1.1 [1.0-1.3] | 0.12 |
| PCT per 10 ng/ml increase | 181 | 1.1 [1.0-1.2] | 0.08 |
| Bilirubin per mg/dL increase | 202 | 1.1 [1.0-1.1] | **<0.001** |
| Creatinine per mg/dL increase | 202 | 1.1 [1.0-1.2] | **<0.001** |
|  |  |  |  |
| **Mycological findings** |  |  |  |
| Serum GM (ODI) | 192 | 1.2 [1.0-1.4] | **<0.001** |
| BAL GM (ODI) | 152 | 1.0 [1.0-1.1] | 0.17 |
| Β-D-Glucan per 500 pg/mL | 195 | 1.2 [1.0-1.4] | **0.02** |
| Asp-PCR | 68 | 1.0 [0.5-2.0] | 0.96 |
| Asp-LFD | 61 | 1.1 [0.5-2.4] | 0.80 |
| BAL-Culture | 199 | 0.6 [0.2-0.9] | **0.02** |
|  |  |  |  |
| **Immunosuppression** |  |  |  |
| Neutropenia | 202 | 1.1 [0.6-2.0] | 0.66 |
| Haematological Malignancy | 202 | 1.0 [0.6-1.7] | 0.94 |
| Corticoids | 202 | 1.2 [0.7-1.8] | 0.52 |
|  |  |  |  |
| **ICU-characteristics** |  |  |  |
| ICU admission | 202 | 4.0 [2.0-8.4] | **<0.001** |
| APACHE | 202 | 1.2 [1.2-1.3] | **<0.001** |

**Supplementary Table 4:** Univariable Cox regression analysis assessing hazard ratios (HR) for predictors of 30-day mortality. The table includes hazard ratios (HR) with 95% confidence intervals (CI) and p-values for each variable. HR - Hazard Ratio; CI - Confidence Interval; IPA - Invasive Pulmonary Aspergillosis; PCP - *Pneumocystis jirovecii* Pneumonia; BMI - body mass index, CRP - C-reactive protein; PCT procalcitonin; GM – galactomannan; ODI - optical density index; BAL - bronchoalveolar lavage; Asp-PCR - *Aspergillus* polymerase chain reaction; Asp-LFD - *Aspergillus* lateral flow device; APACHE - Acute Physiology and Chronic Health Evaluation; ICU - Intensive Care Unit.
